# APP Overexpression Causes Aβ-Independent Neuronal Death through Intrinsic Apoptosis Pathway

**DOI:** 10.1523/ENEURO.0150-16.2016

**Published:** 2016-08-01

**Authors:** Ning Cheng, Song Jiao, Ankita Gumaste, Li Bai, Leonardo Belluscio

**Affiliations:** Developmental Neural Plasticity Section, National Institute of Neurological Disorders and Stroke, National Institutes of Health, Bethesda, Maryland 20892

**Keywords:** amyloid precursor protein, apoptosis, neurodegeneration, olfactory

## Abstract

Accumulation of amyloid-β (Aβ) peptide in the brain is a central hallmark of Alzheimer’s disease (AD) and is thought to be the cause of the observed neurodegeneration. Many animal models have been generated that overproduce Aβ yet do not exhibit clear neuronal loss, questioning this Aβ hypothesis. We previously developed an *in vivo* mouse model that expresses a humanized amyloid precursor protein (hAPP) in olfactory sensory neurons (OSNs) showing robust apoptosis and olfactory dysfunction by 3 weeks of age, which is consistent with early OSN loss and smell deficits, as observed in AD patients. Here we show, by deleting the β-site APP cleaving enzyme 1 (BACE1) in two distinct transgenic mouse models, that hAPP-induced apoptosis of OSNs is Aβ independent and remains cell autonomous. In addition, we reveal that the intrinsic apoptosis pathway is responsible for hAPP-induced OSN death, as marked by mitochondrial damage and caspase-9 activation. Given that hAPP expression causes OSN apoptosis despite the absence of BACE1, we propose that Aβ is not the sole cause of hAPP-induced neurodegeneration and that the early loss of olfactory function in AD may be based on a cell-autonomous mechanism, which could mark an early phase of AD, prior to Aβ accumulation. Thus, the olfactory system could serve as an important new platform to study the development of AD, providing unique insight for both early diagnosis and intervention.

## Significance Statement

Understanding the basis of neuronal loss in Alzheimer’s disease (AD) is fundamental to understanding disease progression, and developing new diagnostic and treatment strategies. Olfactory loss occurs early in AD and offers a unique perspective into the neurodegenerative process. Here we show that overexpression of hAPP can cause early apoptosis of olfactory sensory neurons in the absence of the amyloid-β peptide and in a cell-autonomous manner. We further demonstrate the activation of the intrinsic apoptosis pathway in hAPP-expressing neurons, highlighting a potential role for cellular stress and mitochondrial regulatory factors in mediating this early neuronal loss.

## Introduction

More than 5 million Americans have Alzheimer’s disease (AD), yet there are no effective treatments (Alzheimer's Association, 2013). The amyloid hypothesis posits that the widespread neurodegeneration found in AD patients is caused by cerebral accumulation of the cytotoxic amyloid-β (Aβ) peptide, which is derived from the amyloid precursor protein (APP) and forms plaques (Hardy and Selkoe, 2002). While this hypothesis has gained strong support from studies in both human patients and animals models (Hardy and Selkoe, 2002), recent findings have identified Aβ-independent mechanisms that may also contribute to AD-related neurodegeneration (Pimplikar et al., 2010; Winton et al., 2011). Resolving this issue has been difficult as most transgenic models that overproduce Aβ to generate cerebral deposits poorly mimic the neuronal loss found in AD patients (Wirths and Bayer, 2010). Thus, the question remains: is Aβ the primary source of neurodegeneration in AD?

Olfactory sensory neuron (OSN) loss and smell deficits occur early in AD (Talamo et al., 1989; Bacon et al., 1998). We previously established an olfactory model of AD-related neurodegeneration in which we expressed a humanized APP gene (hAPP), containing both the Swedish and Indiana familial AD mutations, under the control of olfactory-specific promoters that target expression in either mature (OMP-hAPP line) or immature OSNs (Gγ8-hAPP line; Cheng et al., 2011). Importantly, we observed large-scale apoptosis of OSNs in both lines by 3 weeks of age with no detectable extracellular Aβ deposits (Cheng et al., 2011, 2013; Saar et al., 2015). This finding raised questions about the participation of Aβ in APP-induced neurodegeneration while presenting us with a unique model to explore the underlying mechanism, since OSN loss is robust and can be observed relatively early. Thus, we sought to determine whether Aβ peptide is required for APP-induced OSN cell death and which apoptosis pathway mediates the induced neurodegeneration.

## Materials and Methods

### Transgenic lines

We utilized the tetracycline-transactivation system (tet-off), allowing spatial–temporal control of transgene expression (Gossen and Bujard, 1992; Lewandoski, 2001). The TetO-hAPP line contains the hAPP transgene (humanized Aβ-domain with familial AD mutations: KM570, 571NL “Swedish,” and V617F “Indiana”; Jankowsky et al., 2005). The OMP-tTA line expresses the tetracycline transactivator in mature OSNs (Yu et al., 2004; Nguyen et al., 2007). The OMP-tTA line was crossed with the tetO-hAPP line to generate a line that selectively expresses hAPP in mature OSNs driven by the OMP promoter (OMP-hAPP line). Similarly, the Camk2a-tTA line expresses the tetracycline transactivator under the control of calcium/calmodulin-dependent kinase II (Camk2a) promoter (Mayford et al., 1996). The Camk2a-tTA line was crossed with the tetO-hAPP line to generate a line (the Camk2a-hAPP line) that expresses hAPP in many neuronal types in both the brain and the olfactory epithelium (OE). Genotyping was performed to recognize mutants containing both the tTA and tetO transgenes. Littermates containing no transgene, only the tetO transgene, or only the tTA transgene were examined, and the results were indistinguishable. In most cases, littermates containing the only tetO transgene were selected as controls. Camk2a-hAPP lines were crossed with the null β-site APP cleaving enzyme 1 (BACE1^−/−^) line to generate Camk2a-hAPP/BACE1^+/−^ and Camk2a-hAPP/BACE1^−/−^ lines. A similar breeding scheme was used to generate OMP-hAPP/BACE1^+/−^ and OMP-hAPP/BACE1^−/−^ lines. OMP-hAPP lines were also directly crossed with OMP-GFP reporter mice to generate OMP-hAPP/OMP-GFP^+/−^ mutant mice and tetO-hAPP/OMP-GFP^+/−^ controls. Both sexes were used for experiments, and mice were mixed (129 × C57BL/6) background as defined by The Jackson Laboratory [stock #006667, #007052, #017754, and #003010 (ordered before 2012)]. Each line was backcrossed at least two times with C57BL/6 mice (stock #000664) then maintained by interbreeding heterozygous animals.

### PCR primers

The following PCR primers were used for genotyping: OMP-tTA: 5' GGTTGCGTATTGGAAGATCAAGAGC 3'; 5' GAGGAGCAGCTAGAAGAATGTCCC 3'; tetO-hAPP: 5' CCGAGATCTCTGAAGTGAAGATGGATG 3'; 5' CCAAGCCTAGACCACGAGAATGC 3'; Camk2a-tTA: 5' CGCTGTGGGGCATTTTACTTTAG 3'; 5' CATGTCCAGATCGAAATCGTC 3'; BACE: 5' AGG CAG CTT TGT GGA GAT GGT G 3' (wild type); 5' CGG GAA ATG GAA AGG CTA CTC C 3' (common); and 5' TGG ATG TGG AAT GTG TGC GAG 3' (mutant).

### Immunohistochemistry

Fluorescence immunohistochemistry was performed as previously described (Cheng et al, 2011). The following primary antibodies were used: hAPP, 1:1000 (6E10, Covance); OMP, 1:5000 (Wako); Gap43, 1:1000 (Novus Biologicals); cleaved caspase-3, 1:1000 (Cell Signaling Technology); Camk2a, 1:1000 (Abcam); and cleaved caspase-9, 1:200 (Cell Signaling Technology). Sections were examined using confocal microscopy (LSM510 microscope, Zeiss).

### ELISA of Aβ

Colorimetric sandwich ELISA kits with antibodies against human Aβ_42_ (Invitrogen) were used. Acutely dissected olfactory epithelium tissue was homogenized and centrifuged. Supernatant was loaded on ELISA plate. Assay was performed according to manufacturer's manual with all standards and samples measured in duplicate. *N* represents numbers of animals.

### Western blot analysis

Olfactory epithelia and brains of 3-week-old mice were dissected and homogenized in RIPA buffer (Sigma-Aldrich) with protease inhibitors (Calbiochem). The protein extract was separated by SDS-PAGE, transferred to nitrocellulose membrane, incubated with antibodies then visualized using a chemiluminescent detection kit (ECL Plus, GE Healthcare). The following primary antibodies were used: 6E10, 1:2000 (Covance) to detect full-length hAPP; anti-APP C-terminal antibody A8717, 1:11,000 (Sigma-Aldrich); anti-cleaved caspase-3, 1:1000; anti-cleaved caspase-9, 1:500; anti-full-length caspase-3, 1:500; anti-full-length caspase-9, 1:500 (all from Cell Signaling Technology); anti-Bax, 1:500; and anti-β-actin, 1:4000 (both from Sigma-Aldrich).

### Cell counting

Caspase-3- and caspase-9-positive cells in the septal epithelium spanning dorsal to ventral zones were counted manually using signal intensity and size threshold. Images of four to six sample sections were taken from each animal representing the anterior, middle, and posterior parts of the turbinates. Cell counts were expressed as the number of cells per millimeter in the septal epithelium. *N* represents numbers of animals.

### Electron microscopy

A previously established protocol (Tao-Cheng et al., 2006) was followed. Mice were perfused transcardially with 2% PFA and 2% glutaraldehyde in 0.1N cacodylate buffer. Sections of OE were prepared by the Electron Microscopy Facility of the National Institute of Neurological Disorders and Stroke, and examined with an electron microscope (1200EX II, JEOL).

### Mitochondrial membrane potential assay

Mitochondrial membrane potential is monitored by the staining of tetramethylrhodamine ethyl ester (TMRE; Life Technologies), which is a cell-permeable and negatively charged dye that accumulates in active mitochondria in a potential-dependent manner. TMRE was prepared in DMSO for 1 mm stock and diluted to a 100 nm working solution in Ringer’s solution (Hospira). Fresh olfactory epithelium tissues were dissected from 4-week-old mice and sectioned at 200 μm thickness in ice-cold Ringer’s solution on a Leica Vibratome. Acute OE sections were incubated in TMRE working solution at 37°C for 30 min, gently rinsed in Ringer’s solution, and then immediately imaged under a fluorescence microscope.

### Statistical analysis

A Student’s *t* test was performed to test statistical significance, assuming two-tailed distribution and two-sample unequal variance. All values are reported as the mean ± SD. The *p* values (*p < 0.05 and **p < 0.001) are also indicated in the corresponding figure legend.

## Results

**Figure 1.**
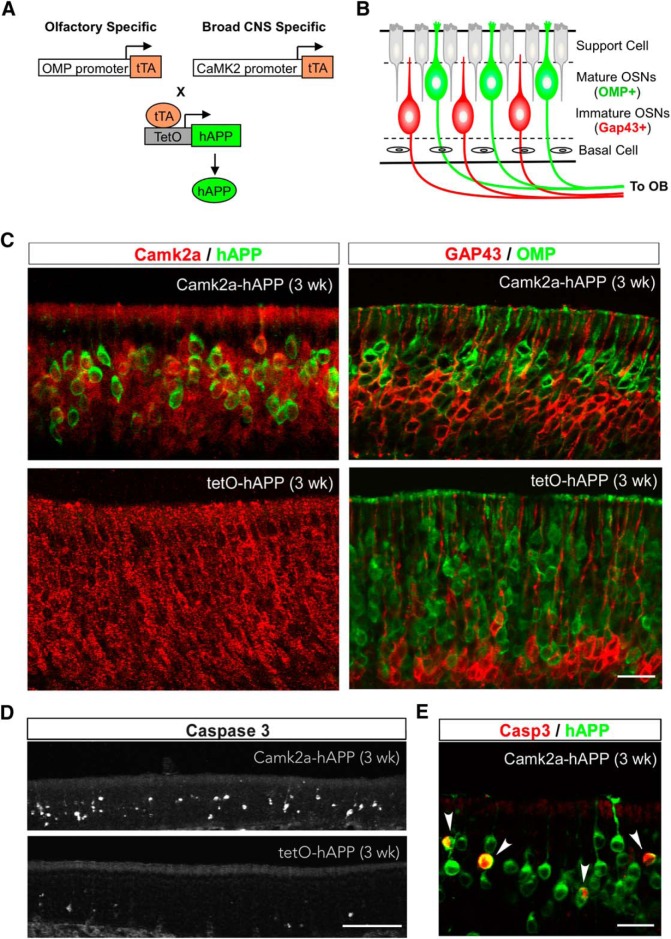
OSN apoptosis in Camk2a-hAPP mice. ***A,*** Strategies to generate mutant lines with olfactory-specific or broad CNS-specific overexpression of hAPP by using OMP or Camk2a promoter, respectively, and the tTA-TetO system. ***B,*** Diagram showing the organization of the OE, with markers for mature and immature OSNs highlighted. ***C***, Left panels, Camk2a (red) and hAPP (green) immunohistochemical signal in the olfactory epithelium from 3-week-old Camk2a-hAPP and tetO-hAPP mice, respectively. Camk2a was broadly expressed in OSNs and mostly colocalized with hAPP in Camk2a-hAPP mice. Right panels, GAP43 (immature OSN marker, red) and OMP (mature OSN marker, green) immunohistochemical signal in the epithelium. Note that Camk2a-hAPP animals had less mature OSNs and thinner epithelia than controls. ***D***, ***E***, The 3-week-old Camk2a-hAPP animal had many more cleaved caspase-3-positive cells in the epithelium than the control animal (***D***), which colocalized in hAPP-expressing neurons (***E***, arrowheads). Scale bars: ***C***, ***E***, 20 µm; ***D***, 100 µm.

**Figure 2.**
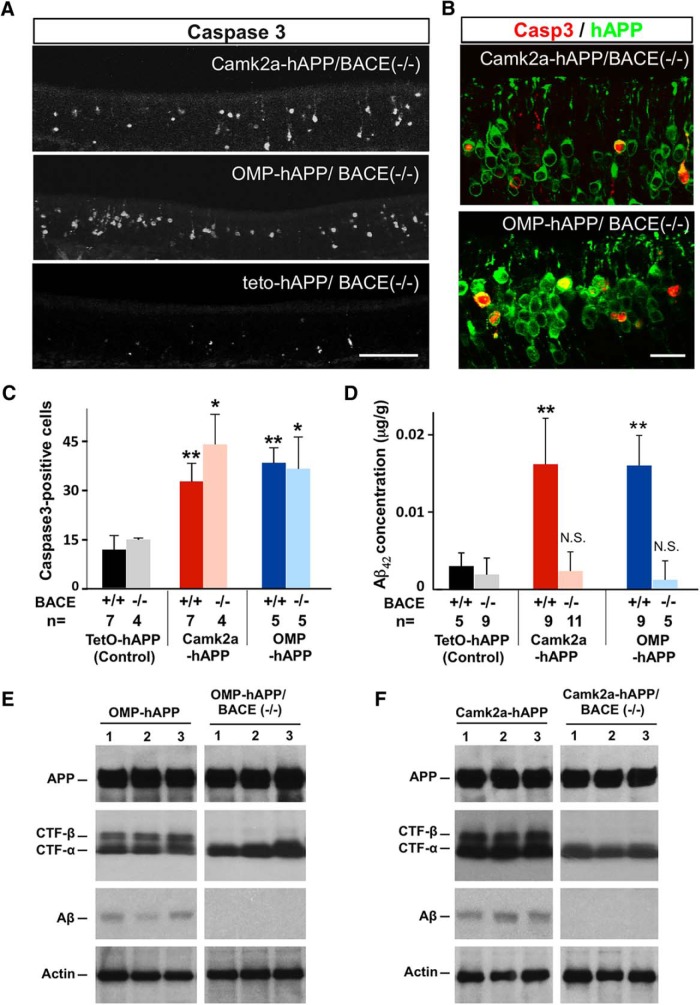
hAPP-induced apoptosis persisted in a BACE^−/−^ background. ***A***, Cleaved caspase-3 signal in 3-week-old Camk2a-hAPP/BACE^−/−^, OMP-hAPP/BACE^−/−^, and control animals. ***B***, The mutants had many more OSNs with caspase-3 signal than controls, which colocalized with the hAPP signal. ***C***, Quantification of caspase-3-positive cells showed that hAPP-expressing lines had significantly more dying cells than the control lines, regardless of BACE genotype (Camk2a-hAPP, 32.8 ± 5.5, *n* = 7; and OMP-hAPP, 38.5 ± 4.6, *n* = 5, compared with tetO-hAPP, 11.7 ± 4.3, *n* = 7; while Camk2a-hAPP/BACE^−/−^, 44.1 ± 9.2, *n* = 4, and OMP-hAPP/BACE^−/−^, 36.3 ± 9.7, *n* = 5, compared with tetO-hAPP/BACE^−/−^, 14.8 ± 0.2, *n* = 4). ***D***, ELISA on OE tissue shows very low levels of Aβ_42_ concentration in hAPP-expressing mutants with a null BACE background, not significantly different from the levels found in the control lines. ***E***, ***F***, Western blots confirming similar levels of full-length hAPP protein expression in the OEs from OMP-hAPP mice and OMP-hAPP/BACE^−/−^ mice (***E***), as well as Camk2a-hAPP mice and Camk2a-hAPP/BACE^−/−^ mice (***F***; individual animals in each lane). Actin was used as a loading control. The expression of α-CTF was also detected across all mutant genotypes, while β-CTF was absent in both the OMP-hAPP/BACE^−/−^ mice and Camk2a-hAPP/BACE^−/−^ mice, consistent with a loss in BACE activity. In addition, Aβ peptide is found in OEs from OMP-hAPP and Camk2a-hAPP mice, but is not detectable in OMP-hAPP/BACE^−/−^ and Camk2a-hAPP/BACE^−/−^ mice, further confirming the lack of BACE activity. All values are reported as the mean ± SD. Scale bars: ***A***, 100 µm; ***B***, 20 µm. *p < 0.01, **p < 0.001.

To extend our previous findings showing hAPP-induced OSN loss through olfactory-specific expression, we used a third mouse model in which hAPP is expressed using the Camk2a promotor, which is broadly active in many excitatory neurons (Mayford et al., 1996), including both mature and immature OSNs (Wei et al., 1998). By crossing Camk2a-tTA mice (Mayford et al., 1996) with tetO-hAPP mice (Jankowsky et al., 2005), we generated a mutant line (Camk2a-hAPP) to determine whether OSN cell death occurs earlier than neuronal loss in other brain regions (Fig. 1*A*,*B*). A previous study using the same Camk2a-hAPP line reported amyloid deposits in many brain areas by 2 months of age, but no active apoptosis (Jankowsky et al., 2005). Here we show broad colocalization of hAPP immunohistochemical signal and endogenous Camk2a signal in OSNs from 3-week-old mutant animals (Fig. 1*C*), which also exhibit a thinner OE and a clear reduction in mature OSNs compared to controls **(** Fig. 1*C***)**. Importantly, analysis with antibody against cleaved caspase-3 revealed that Camk2a-hAPP animals had significantly more apoptotic cells in the OE than controls (Figs. 1*D*, 2*C* ; *p* = 6 × 10^−6^), which visibly colocalized with hAPP expression (Fig. 1*E*). These results demonstrate a clear increase in OSN cell death in Camk2a-hAPP mice, which is similar to what was previously observed in OMP-hAPP mice (Cheng et al., 2011).

To test whether accumulation of Aβ peptide was the direct cause of OSN cell death in Camk2a-hAPP mice, we crossed them with BACE1^−/−^ mice (Cai et al., 2001) to generate Camk2a-hAPP/BACE1^−/−^ compound mutant mice. Since Aβ peptide is generated by the sequential proteolytic cleavage of APP via β- and γ-secretases (O'Brien and Wong, 2011), and BACE1 is the major β-secretase used by neurons (Cai et al., 2001), Aβ peptide should be nearly absent in the Camk2a-hAPP/BACE1^−/−^ mice. Using an ELISA, we quantified Aβ_42_ (amyloidogenic APP fragment) in OE tissue from 3-week-old animals and indeed found extremely low peptide levels in Camk2a-hAPP/BACE1^−/−^ animals, similar to both the tetO-hAPP and tetO-hAPP/BACE1^−/−^ control groups, and in sharp contrast to the high levels found in Camk2a-hAPP/BACE1^+/+^ mice (Fig. 2*D*; *p* < 0.001). Interestingly, despite the loss of Aβ peptide, we still observed a large number of caspase-3-positive cells in the Camk2a-hAPP/BACE1^−/−^ mouse epithelium (Fig. 2*A*), similar to those observed in Camk2a-hAPP/BACE1^+/+^ animals (*P* = 0.08), and significantly greater than those in controls (Fig. 2*C*, *p* = 0.008). Similar results were observed when we crossed the OMP-hAPP line from our previous study into the BACE1^−/−^ background and compared them to controls (Fig. 2*C*; *P* = 0.008), indicating that neither BACE1 activity nor increased Aβ levels were necessary for hAPP-induced apoptosis of OSNs. Moreover, the caspase-3 signal clearly colocalized with hAPP expression in all mutant lines (Fig. 2*B*), further demonstrating the autonomous nature of the cell loss (Cheng et al., 2011). To further confirm the absence of BACE1 activity in the compound mutant lines, we examined the expression levels of the C-terminal fragments of hAPP cleavage using Western blot, and found that β-C-terminal fragment (β-CTF) was absent in the BACE1^−/−^ background, while the expression levels of the full-length hAPP and the α-C-terminal fragment (α-CTF) showed little change (Fig. 2*E*,*F*).

**Figure 3.**
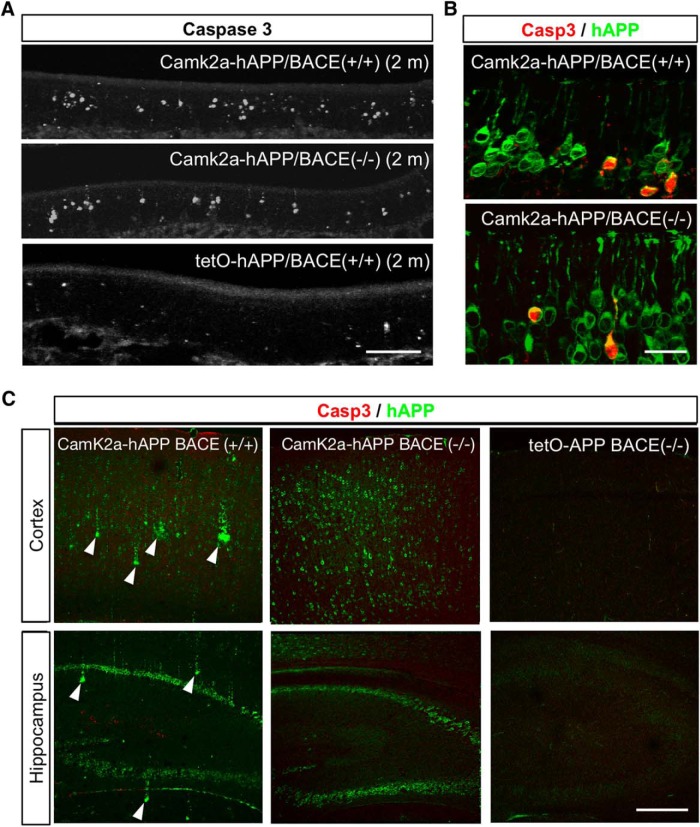
Cell death in the olfactory epithelium continued into adulthood without Aβ deposits. ***A***, Cleaved caspase-3 signal in the OE of 2-month-old Camk2a-hAPP/BACE^−/−^, OMP-hAPP/BACE^−/−^, and control animals. ***B***, At this age, the mutants still had many more OSNs with caspase-3 signal than controls, which colocalized with hAPP signal. ***C***, Amyloid deposits developed in the cortex and hippocampus of 2-month-old Camk2a-hAPP/BACE^+/+^ mice (arrowheads), but not Camk2a-hAPP/BACE^−/−^ or tetO-hAPP/BACE^−/−^ mice. Cleaved caspase-3 signal was not elevated in the cortex or hippocampus of any genotype at either 3 weeks of age (data not shown) or 2 months of age. Scale bars: ***A***, 100 µm; ***B***, 20 µm; ***C***, 200 µm.

To determine whether hAPP-induced neurodegeneration of OSNs continued into adulthood, we also examined OEs from 2-month-old mice and found that increased levels of caspase-3-positive cells persisted in both Camk2a-hAPP/BACE1^+/+^ and Camk2a-hAPP/BACE1^−/−^ mice (Fig. 3*A*,*B*). Interestingly, we observed the formation of Aβ deposits in cortical and hippocampal areas of 2-month-old Camk2a-hAPP/BACE1^+/+^ animals, as previously reported (Jankowsky et al., 2005), but found them absent in Camk2a-hAPP/BACE1^−/−^ animals (Fig. 3*C*), further demonstrating that Aβ was not present in the BACE-null background and therefore could not be the basis of increased OSN apoptosis. In addition, despite the widespread expression of hAPP in higher brain regions of the Camk2a-hAPP animals, there was no increase in caspase-3 signal in these areas either at 3 weeks of age (data not shown) or 2 months of age (Fig. 3*C*; Jankowsky et al., 2005; Wirths and Bayer, 2010), suggesting that the olfactory system exhibits a clear phenotype through hAPP-induced cell death that is measurable much earlier than corresponding changes in cortical regions. This difference in timing both underscores the utility of an olfactory model, and may be the basis of the OSN loss and early smell deficits reported in AD patients (Talamo et al., 1989; Bacon et al., 1998).

**Figure 4.**
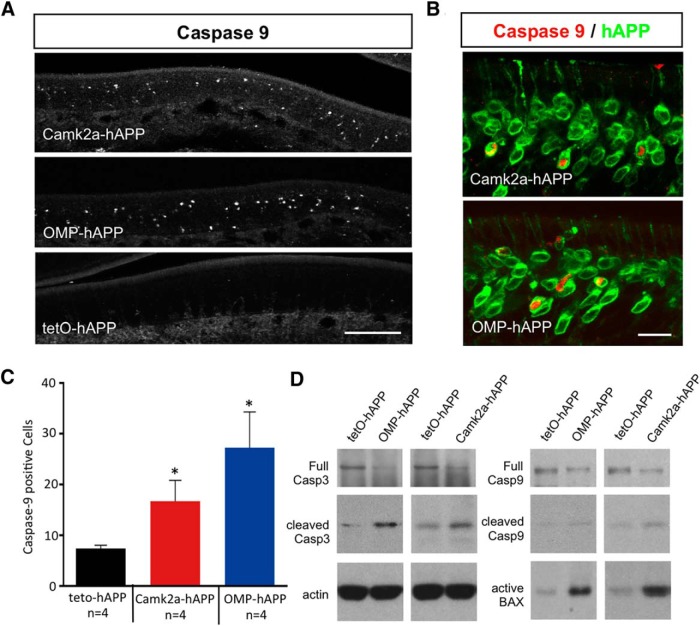
Active caspase-9 expression in hAPP-expressing OSNs of both OMP-hAPP and Camk2a-hAPP mice. ***A***, ***B***, Cleaved caspase-9 signal in the OE of 3-week-old OMP-hAPP, Camk2a-hAPP, and control animals (***A***) showed OSNs that were positive for caspase-9 signal and clearly colocalized with hAPP signal shown in both mutant lines (***B***). ***C***, Quantification of caspase-9-positive cells showed a significant increase in both hAPP-expressing lines compared with the controls (Camk2a-hAPP, 16.7 ± 4.1, *n* = 4; and OMP-hAPP, 27.3 ± 7.0, *n* = 4; compared with tetO-hAPP, 7.4 ± 0.6, *n* = 4). ***D***, In addition, relative expression levels of the apoptosis markers, active caspase-3, caspase-9, and BAX were all increased in the mutant lines compared with controls, while full-length caspase-3 and caspase-9 levels showed a small reduction in mutant lines compared with control, which is consistent with an increased conversion to their cleaved active forms. Scale bars: ***A***, 100 µm; ***B***, 20 µm. *p < 0.05.

Since active apoptosis is a unique feature of this model, we next sought to determine which of the apoptosis pathways (Elmore, 2007; Tait and Green, 2010) was responsible for triggering the hAPP-induced OSN loss. We first examined the expression of several initiator caspases in the OE of both OMP-hAPP and Camk2a-hAPP mice, and, while we did not detect any cleaved caspase-8 in the OE (data not shown), we found many cells positive for cleaved caspase-9 in both mutant lines (Fig. 4*A*). We showed that these cleaved caspase-9-positive cells directly colocalized with hAPP-expressing OSNs (Fig. 4*B*) and that their numbers were significantly higher in both mutant lines compared with controls (Camk2a-hAPP, *p* = 0.02; OMP-hAPP, *p* = 0.01; Fig. 4*C*). Western blot analysis showed similar results indicating an increase in both cleaved caspase-3 and caspase-9 protein levels in OE tissue from both mutant lines, while full-length caspase-3 and caspase-9 levels showed a small reduction compared with controls, which is consistent with their active conversion to the cleaved form (Fig. 4*D*). Interestingly, we also revealed a striking increase in BCL-2-associated X (BAX) protein in both mutant lines, which is typically associated with mitochondrial damage and linked to caspase-9 cleavage via the intrinsic apoptosis pathway (Elmore, 2007; Tait and Green, 2010; Fig. 4*D*).

**Figure 5.**
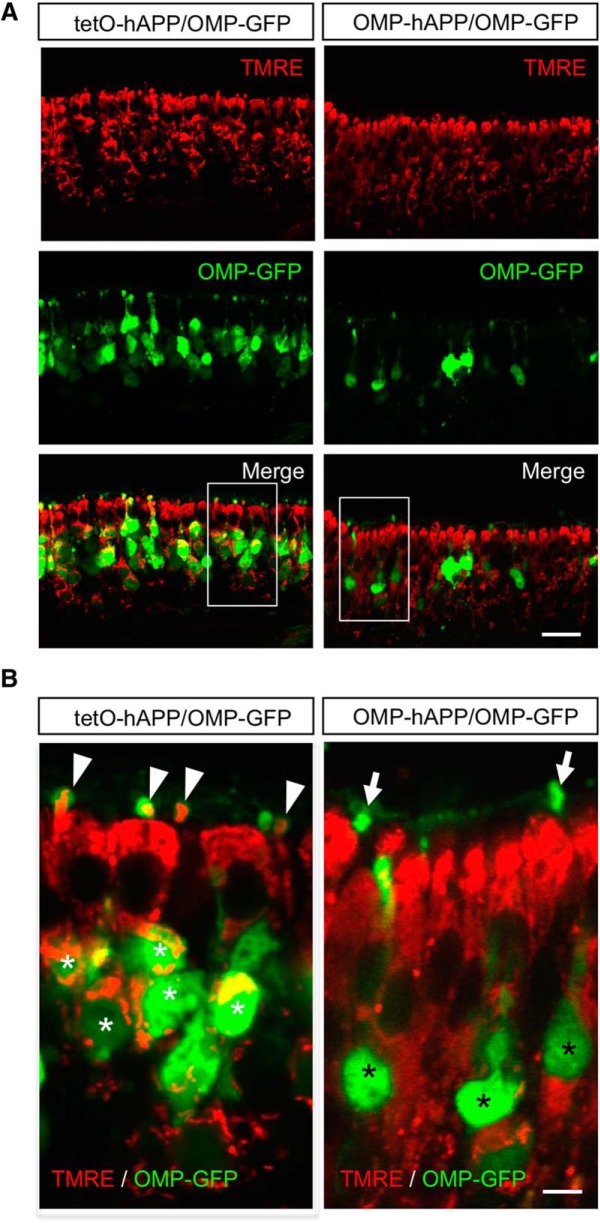
Vital-dye staining indicates dysfunctional mitochondria in hAPP-expressing OSNs. ***A***, Fluorescent images of olfactory epithelium from OMP-hAPP mutant mice (right panels) and tetO-hAPP controls (left panels) comparing *in vivo* mitochondrial staining via TMRE indicator (red) in OMP-GFP-positive OSNs (green). ***B***, Close-up of boxed regions in ***A*** showing that OSNs from control mice (left) contain live mitochondria (white asterisks) that are also detectable in the OSN dendritic knobs (arrowhead). By comparison, OSNs from mutant mice (right) show little colocalization with TMRE signal both in cell bodies (black asterisks) and in dendritic knobs (arrows). Scale bars: ***A***, 20 µm; ***B***, 5 µm.

**Figure 6.**
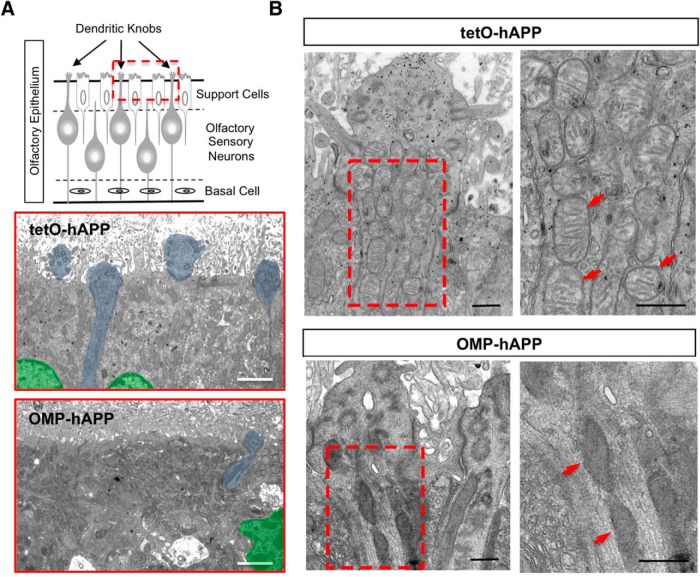
Ultrastructural imaging of OE shows damaged mitochondria in dendritic knobs of hAPP-expressing OSNs. ***A***, Schematic of OE depicting the superficial sustentacular support cells lining the apical surface with OSN dendritic knobs protruding between them into the lumen. Electron micrographs corresponding to the OE apical region (red boxed region in schematic) with tetO-hAPP control (middle) showing three support cells, two with distinct nuclei (shaded green), and portions of four OSN dendritic knobs (shaded blue) protruding into the lumen among the fragmented cilia, while an OMP-hAPP mutant (bottom) shows disrupted support cell organization (green nucleus) and very few OSN dendritic knobs (one shaded blue). ***B***, Comparison of an OSN dendritic knob from tetO-hAPP control (top panels) and OMP-hAPP mutant (bottom panels) reveals a clear alteration in the mitochondrial morphology of OMP-hAPP animals, which appear dark with indistinct features compared with the healthy appearance of mitochondria in the control animals showing clear cisternae. The panels on the right correspond to the boxed regions on the left panels. Arrowheads point to mitochondria. Scale bars: ***A***, 2 µm; ***B***, 500 nm.

**Figure 7.**
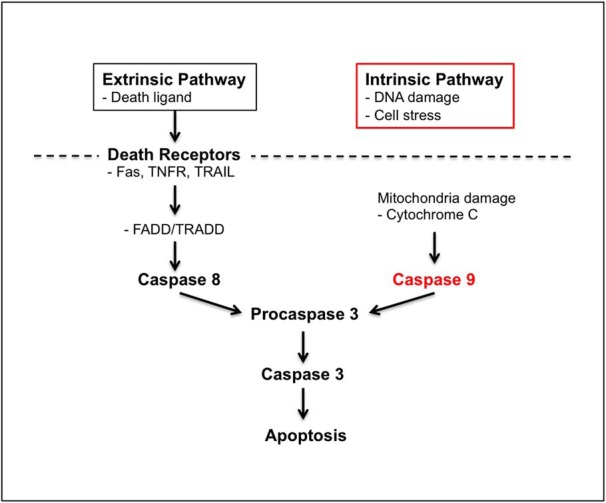
hAPP expression activates the intrinsic apoptosis pathway in OSNs. Schematic showing the key steps in both extrinsic and intrinsic pathways of apoptosis. Both intrinsic and extrinsic apoptosis pathways can lead to cell death by activating common late stage factors, such as caspase-3 but may be triggered through distinct mechanisms. The extrinsic pathway is typically activated via death receptors in the plasma membrane [e.g., Fas, TNFR (tumor necrosis factor receptor), TRAIL (tumor necrosis factor-related apoptosis-inducing ligand)], which activate FADD (FAS-associated death domain)/TRADD (Tumor necrosis factor receptor type 1-associated death domain), leading to caspase-8-mediated activation of the late-stage apoptosis pathway. The intrinsic pathway can be triggered by cell-autonomous factors (e.g., DNA damage, cell stress) possibly involving mitochondrial dysfunction and activation of caspase-9, which can activate caspase-3 followed by apoptosis. The clear activation of caspase-9 in hAPP-expressing OSNs both in OMP-hAPP and Camk2a-hAPP mutant mouse lines combined with the overt mitochondrial changes strongly indicate that hAPP-induced apoptosis is mediated by the intrinsic pathway.

Thus, to examine the state of mitochondria in hAPP-expressing OSNs, we used TMRE, a vital-dye stain for mitochondria applied directly to OE tissue. Since TMRE labeling is dependent upon mitochondrial membrane potential, it can be used as a marker of functional mitochondria. Given that TMRE is sensitive to fixation, we crossed both the OMP-hAPP mutant mice and tetO-hAPP controls with the OMP-GFP reporter line (Potter et al., 2001), which enabled us to directly identify OMP-hAPP-expressing OSNs in live tissue via the GFP label. In Figure 5, we show that in tetO-hAPP control mice TMRE effectively labels mitochondria within the mature OSNs (OMP-GFP positive) and also in sustentacular cells. By comparison, TMRE applied to OE tissue from OMP-hAPP mice shows labeling in sustentacular cells but very little signal in hAPP-expressing OSNs (identified by OMP-GFP), suggesting dysfunctional mitochondria. We next performed electron microscopy on OE from both mutant and control animals to examine OSN mitochondria at higher resolution and observed a clear difference in mitochondrial morphology. Mitochondria in mutant OSNs showed a darker matrix and indistinct cristae, which was in sharp contrast to the healthy appearance of mitochondria in the control OSNs (Fig. 6). Together, these data suggest that hAPP-induced cell death of OSNs is initiated in a cell-autonomous manner and mediated by the intrinsic apoptosis pathway (Fig. 7).

## Discussion

Our olfactory studies have presented three transgenic lines (OMP-hAPP, Gγ8-hAPP, and Camk2a-hAPP), all of which show visible APP-induced apoptosis of OSNs by 3 weeks of age. Since the Camk2a promoter drives hAPP expression both in OSNs and throughout the brain, the consistent early emergence of olfactory phenotypes suggests that OSNs are more sensitive to the detrimental effects of hAPP than other neuronal types. If so, this would provide a scientific basis for olfactory dysfunction occurring early in AD (Talamo et al., 1989; Bacon et al., 1998; Hawkes, 2003; Doty, 2009; Arnold et al., 2010) and present a clear advantage to using OSN for uncovering these early-stage mechanisms. In addition, their increased sensitivity to neurodegenerative factors makes OSNs an attractive candidate for use in screening assays.

It has been generally accepted that apoptosis contributes to neurodegeneration in AD (Behl, 2000; Shimohama, 2000), as evidence of apoptotic cell death has been reported in patients (Behl, 2000; Shimohama, 2000). However, the mechanisms remain unknown, in part due to the many animal models of AD that do not display active neurodegeneration (Wirths and Bayer, 2010). Studies of apoptosis in both physiological and pathological conditions have shown that it is a highly sophisticated process, with two main pathways, the intrinsic or mitochondrial pathway and the extrinsic or death receptor pathway (Elmore, 2007; Tait and Green, 2010). The intrinsic pathway involves the signaling cascade of mitochondrial damage, the release of cytochrome *c*, and the activation of caspase-9, while the extrinsic pathway involves transmembrane receptor-mediated interactions and the activation of caspase-8. Both pathways converge on the same execution cascade initiated by the cleavage of caspase-3 (Elmore, 2007; Tait and Green, 2010). Previous studies have shown evidence of mitochondrial damage in AD patients (Lin and Beal, 2006), implicating activation of the intrinsic pathway. Using our olfactory models in which large-scale neurodegeneration is readily observable, we show here that the intrinsic apoptosis pathway is clearly activated in OSNs overexpressing hAPP, supporting this link and further suggesting that targeting this pathway may hold some therapeutic value.

In the past several decades, significant effort has centered on eliminating Aβ plaques or reducing Aβ levels as a general strategy for combating AD. Unfortunately, therapeutic development based on this approach either through antibody-based clearance of Aβ (Doody et al., 2014; Salloway et al., 2014) or supressing Aβ production by interfering with BACE1 or γ-secretase activity (Green et al., 2009; Ghosh et al., 2012; Doody et al., 2013), has generally proven ineffective (Karran et al., 2011; Cummings et al., 2014). While there are many reasons why this approach has not been more successful, including compounds that simply fail to perform, it is also possible that Aβ deposits are not the central cause of AD. Indeed, studies have shown that Aβ plaque load does not directly correlate with cognitive function (Engler et al., 2006; Price et al., 2009; Rentz et al., 2010), which is often used as an outcome measure for assessing treatment efficacy. Another possibility is that the progression of AD is such that diagnosis based upon cognitive deficits places the disease beyond a critical threshold for effective intervention through Aβ clearance, making early diagnosis the key factor. Thus, it would be prudent to consider alternative treatment strategies that are not based upon Aβ levels and may be more evident in those aspects of AD that occur prior to cognitive decline, such as olfactory loss.

We have demonstrated that hAPP-induced apoptosis of OSNs occurs independently of BACE1 and Aβ, supporting our assertion that hAPP expression alone can cause widespread cell death of OSNs without the presence of extracellular amyloid deposits (Cheng et al., 2011). Moreover, this finding suggests that Aβ accumulation may not be the sole cause of neuronal death in AD and that there may be an early cell-autonomous phase of the disorder that is independent of Aβ. One possibility involves the APP intracellular C-terminal domain (AICD), which is derived from both the α-CTF and β-CTF, and already has been shown to cause Aβ-independent neurodegeneration (Ghosal et al., 2009). The AICD is also elevated in AD patients and can produce AD-like pathology with cognitive deficits in transgenic mice, again independent of Aβ levels (Ghosal et al., 2009). Interestingly, our olfactory models (OMP-hAPP and Camk2a-hAPP) both show the expected loss of the β-CTF in the BACE1^−/−^ background, but little effect on the α-CTF (Fig. 2*E*,*F*), suggesting that it could be involved in OSN neurodegeneration. Alternatively, given the various other components of APP shown to produce neurodegenerative affects (Ghosal et al., 2009; Nikolaev et al., 2009; Simón et al., 2009; Willem et al., 2015), it is also possible that each piece is involved at different stages of the disease with Aβ crucial to a later stage. Thus, some components may play a larger role during the initial phases of AD, affecting only very sensitive neurons such as OSNs, while other components act later in the disease process, more as catalysts to reinforce and propagate neurodegeneration to less sensitive areas of the brain. While the neurotoxic effects of elevated Aβ are well established (Forman et al., 2004; Querfurth and LaFerla, 2010; Holtzman et al., 2011), our results clearly demonstrate that hAPP-induced neurodegeneration can also occur independent of Aβ, indicating the presence of other pathogenic mechanisms that may be closely linked to early-stage disease and thus provide important insight toward understanding how AD is initiated.

## Synthesis

The decision was a result of the Reviewing Editor Anna Menini and the peer reviewers coming together and discussing their recommendations until a consensus was reached. A fact-based synthesis statement explaining their decision and outlining what is needed to prepare a revision is listed below.

After carefully reading the revised manuscript transferred from <i>The Journal of Neuroscience</i>, I consider it scientifically suitable for publication in <i>eNeuro</i>.
